# High-resolution fabrication of nanopatterns by multistep iterative miniaturization of hot-embossed prestressed polymer films and constrained shrinking

**DOI:** 10.1038/s41378-021-00338-y

**Published:** 2022-02-14

**Authors:** Shady Sayed, P. Ravi Selvaganapathy

**Affiliations:** Department of Mechanical Engineering, McMaster University, Hamilton, ON L8S 4L8 Canada

**Keywords:** Other nanotechnology, Engineering

## Abstract

The fabrication of nanostructures and nanopatterns is of crucial importance in microelectronics, nanofluidics, and the manufacture of biomedical devices and biosensors. However, the creation of nanopatterns by means of conventional nanofabrication techniques such as electron beam lithography is expensive and time-consuming. Here, we develop a multistep miniaturization approach using prestressed polymer films to generate nanopatterns from microscale patterns without the need of complex nanolithography methods. Prestressed polymer films have been used as a miniaturization technique to fabricate features with a smaller size than the initial imprinted features. However, the height of the imprinted features is significantly reduced after the thermal shrinking of the prestressed films due to the shape memory effect of the polymer, and as a result, the topographical features tend to disappear after shrinking. We have developed a miniaturization approach that controls the material flow and maintains the shrunken patterns by applying mechanical constraints during the shrinking process. The combination of hot embossing and constrained shrinking makes it possible to reduce the size of the initial imprinted features even to the nanoscale. The developed multistep miniaturization approach allows using the shrunken pattern as a master for a subsequent miniaturization cycle. Well-defined patterns as small as 100 nm are fabricated, showing a 10-fold reduction in size from the original master. The developed approach also allows the transfer of the shrunken polymeric patterns to a silicon substrate, which can be used as a functional substrate for many applications or directly as a master for nanoimprint lithography.

## Introduction

Nanolithography plays a pivotal role in the integrated circuit (IC) and semiconductor industry as well as for the fabrication of microelectronics and biosensors. The current breakthrough and success of IC manufacturing rely on the ability to continuously miniaturize features and pattern areas on the substrate surface, which can increase the density of transistors while reducing the overall size and dimensions of the chip^1,2^. One scalable approach is the use of nanoimprint lithography (NIL), which can replicate features embedded on high-resolution master molds even down to 10 nm. However, NIL requires the fabrication of high-resolution master patterns, generally using serial writing nanolithography methods such as electron beam or focused ion beam lithography^3–6^. These methods often involve high-cost processes and equipment (usually several millions of dollars), and they are time-consuming, especially for large-area patterning at nanoscale dimensions^7–9^. Thus, developing a simple miniaturization approach that can significantly reduce the feature size from a microscale to a nanoscale can be crucial for the fabrication of nanopatterns for use as master molds at a lower cost without the need of conventional direct write nanolithography techniques.

Prestressed polymer films have been used as a miniaturization approach in the microscale to reduce the size of the original patterns by patterning polymer films, then thermally releasing the prestresses by heating above the glass transition temperature^10,11^. As a result, this approach allows shrinking of the film and hence reducing the size of the imprinted patterns on that shrinkable film^12^. The common patterning methods that have been used to pattern shrinkable films include printing^13–15^, reactive ion etching (RIE)^16,17^, and hot embossing^18^.

Hot embossing is a well-known patterning process that is used to transfer patterns from a master mold into a polymeric substrate. It is one of the important nanolithography methods used to replicate nanofeatures and nanopatterns through a thermal nanoimprint process^19,20^. Although hot embossing has been used to pattern prestressed films, it fails to retain nanoscale patterns after shrinking without losing the topographical features^21^. Due to the shape memory effect, the polymer material relaxes during the shrinking process, causing erasure of the patterns embedded on the polymer film. This results in a significant reduction in the height of the imprinted patterns, which accordingly tend to disappear. We have developed a miniaturization approach that controls the polymer reflow and preserves the topographical features after shrinking by mechanically constraining the film during the shrinking process^22^. The film is mechanically constrained in one direction, which allows the release of the stresses only in the orthogonal direction while minimizing the change in height of the imprinted features. Then the film is constrained in the orthogonal direction to obtain biaxial shrinkage, which finally leads to preserving the topographical features while reducing their size. This constrained shrinking process is scalable down to nanoscale, and a multistep iterative miniaturization process can reduce the size even further.

Here, we report such a multistep miniaturization approach that significantly reduces the pattern size from microscale to nanoscale over several cycles without the need to use conventional nanolithography techniques to fabricate nanopatterns. A combination of hot embossing and constrained shrinking of the prestressed polymer films was developed to transfer the master patterns into the polymer films and allow the retention of shrunken patterns. After shrinking, the miniaturized pattern was used to fabricate a new master, which was then used as a master for the next miniaturization cycle. After three miniaturization steps, the size of the patterns was reduced by a total of 10x, and high-resolution patterns as small as 100 nm were fabricated. This multistep miniaturization approach can potentially be applied to fabricate nanoimprint lithography masters for integrated circuit manufacturing and overcome the challenges associated with fabricating masters at that scale using e-beam lithography.

## Results and discussion

### Multistep miniaturization process

Nanoimprint lithography is a well-established fabrication process that is used to replicate master patterns onto another substrate over a large area with high throughput and low cost. However, the fabrication of master molds required for the nanoimprint lithography process is challenging. Master molds are primarily fabricated by electron beam lithography or focused ion beam techniques, which require expensive equipment (several million $) and a long processing time (tens of hours), particularly for large-area patterning. Thus, developing a fabrication process that can create nanoimprint lithography masters without using such complex processes could be valuable for rapid, low cost, and scalable nanofabrication. One approach that can be pursued is to create low-resolution patterns using scalable and high-throughput fabrication methods such as photolithography and then proportionally miniaturize that microscale pattern into a nanoscale pattern.

**Fig. 1:**
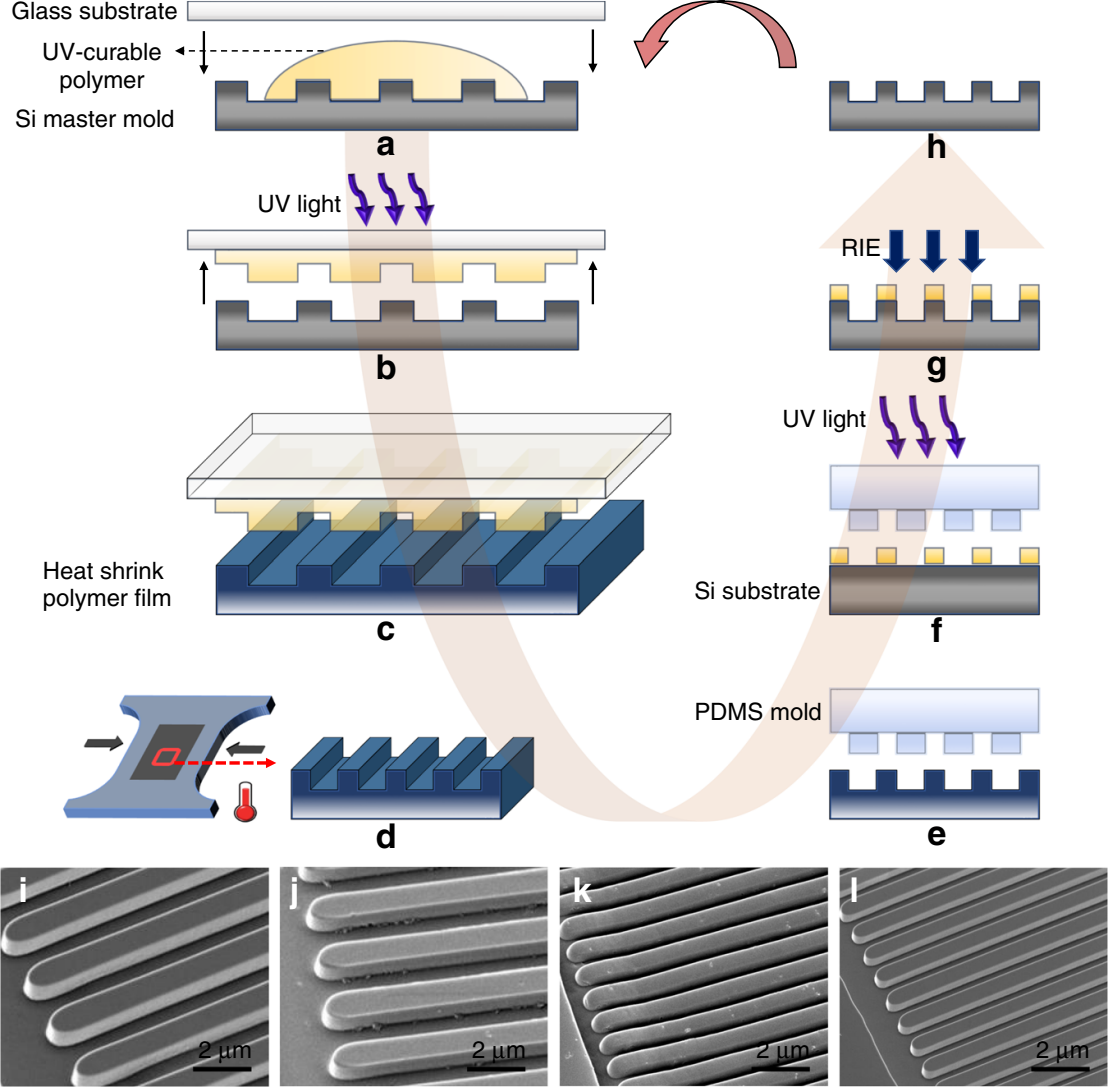
Scheme of the multistep miniaturization approach using prestressed polymer films. **a**, **b** Fabrication of polymer working stamp. **c** Hot embossing the prestressed film. **d** Constrained shrinking. **e** Cast PDMS mold. **f** Soft imprint polymer pattern on Si substrate. **g** RIE Si substrate. **h** Clean patterned Si substrate which is used as a master for the next miniaturization step. SEM images of the results of a complete miniaturization cycle for **i** Si master, **j** imprint on prestressed film, **k** shrunk pattern, **l** new Si master

Following this approach, we have developed a new multistep miniaturization approach based on the constrained shrinking of hot-embossed prestressed polymer films to significantly reduce the size of the initial microscale patterns to the nanoscale while maintaining the topographical features. The developed approach allows the use of the shrunken pattern from a previous step as the master for the next miniaturization cycle, which can be repeated iteratively to achieve the required resolution. A schematic illustration of the multistep miniaturization process is shown in Fig. 1.

First, a Si master mold was fabricated using direct laser writing to create microscale (1 µm) patterns, although they can be also fabricated by photolithography. Then, a polymer working stamp was replicated from the Si master mold to be used directly to imprint the prestressed polymer films. Although the Si master can be used directly for hot embossing, the polymer working stamp was found to be better for demolding of the imprinted film from the stamp (Supplementary Information, section 1)^23,24^. The working stamp was fabricated using a UV-curable polymer that was attached to a glass substrate and cured under a UV light source (Fig. 1a, b). Then, a polystyrene (PS) heat-shrinkable film was imprinted using the fabricated working stamp in a hot embossing process under optimized conditions of molding force, temperature, and time (Fig. 1c). These conditions were optimized to ensure the quality of the imprinted patterns while not affecting the ability of the embossed film to shrink. After hot embossing, the film was carefully demolded from the stamp at a specific separation temperature to avoid damage or deformation of the imprinted patterns during separation.

In order to shrink the patterned film, the film was mechanically constrained in one direction and heated above its glass transition temperature which allowed shrinking only in the orthogonal direction (Fig. 1d). Then, to obtain biaxial shrinkage, the film was constrained in the orthogonal direction and heated. As a result, the size of the patterned features was reduced without losing the topographical features. Next, the shrunken patterns were used to generate a new master to be used for the next miniaturization cycle. After thermal shrinking, the surface of the shrunken film was not completely flat compared to the Si substrate. Moreover, the edges and sidewalls of the shrunken features become more curved due to softening of the polymer during heating. Finally, the aspect ratio of the structures increased, which can result in instability of the line features, especially at the nanoscale. Thus, it was found that fabricating an intermediate Si master was better than using the shrunken PS film directly in terms of obtaining a completely flat substrate surface and decoupling the aspect ratio of the structure from the shrink dynamics, enabling precise structures with vertical sidewalls even at the nanoscale (Supplementary Information, section 4).

In order to generate the new master, polydimethylsiloxane (PDMS) was cast on the shrunken PS film (Fig. 1e) and used to transfer the patterns onto a Si substrate by soft UV imprint lithography (Fig. 1f). Then, the transferred resist pattern was then used as a mask to etch the Si substrate by RIE (Fig. 1g). The RIE was optimized using mixed gas process to successfully transfer patterns onto the Si substrate (Supplementary Information, section 2). Finally, the etched Si substrate was cleaned to remove the remaining resist mask and was used as a master for a next miniaturization cycle (Fig. 1h). Scanning electron microscope (SEM) images of the initial master, imprinted PS film, shrunken film, and new Si master are shown in Fig. 1i, j, k, l, respectively, as an example of the feature evolution through the complete miniaturization cycle. As shown in the figure, the pattern was significantly miniaturized after shrinking, and the topographical features were preserved. In contrast, direct shrinking resulted in losing topographical features where the pattern height was dramatically decreased (Supplementary Information, section 3). It can be noted that the edges of the shrunken pattern became curved (Fig. 1k) compared to the imprinted pattern before shrinking, and the height was maintained (Fig. 1j). However, a pattern with well-defined edges was obtained after RIE of a Si substrate (Fig. 1l), which allows faithful reproduction of the pattern in the next miniaturization step. Due to the introduction of the intermediate Si transfer stamp using the soft UV imprint lithography followed by RIE, the master produced after each step can have independent control over miniaturization in the *X*–*Y* and *Z* directions and enable successful and proportionate miniaturization over a large number of steps. The multistep miniaturization approach allows reducing the size of the initial patterns several times to nanoscale dimensions and generate new masters of smaller size patterns.

**Fig. 2:**
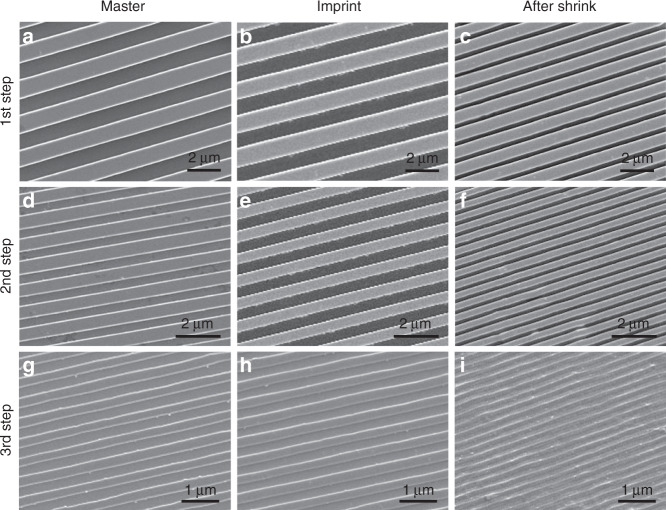
Multistep miniaturization of line-space pattern for three successive cycles. SEM images of **a**, **d**, **g** Si master, **b**, **e**, **h** imprinted prestressed film, and **c**, **f**, **i** shrunk pattern for each miniaturization step. The initial master pattern has line width *w* = 1 μm, spacing *s* = 1 μm. After shrinking of first step *w* = 675 nm, *s* = 320 nm, after second step *w* = 280 nm, *s* = 190 nm, after third step *w* = 130 nm, *s* = 100 nm

The new multistep miniaturization approach can be used as a nanofabrication method that can reduce the size of larger patterns several times down to the nanoscale. This approach demonstrates that the constrained shrinking of hot-embossed patterns on prestressed films can generate well-defined patterns at higher resolution, which can then be used as masters for further miniaturization steps even at 100 nm scale. The scalability of this approach was demonstrated by the miniaturization of a micrometer master pattern into an ~100 nm pattern over three miniaturization steps. The initial master pattern is a line-space pattern with a line width of 1 and 1 µm spacing (Fig. 2a), which was fabricated by using a laser lithographic process. After hot embossing, the imprinted pattern on the PS shrinkable film had same dimensions as the master (Fig. 2b). The patterned film was then miniaturized by applying constrained shrinking which resulted in approximately a 50% reduction in size (Fig. 2c). The shrunken pattern was used to fabricate a new Si master, which was used for the next miniaturization cycle.

Following the procedures described in Fig. 1, the miniaturization process was repeated for three cycles. The initial master and the new fabricated masters that were used for the three miniaturization cycles are shown in Fig. 2a, d, g. The imprinted patterns on prestressed films are shown in Fig. 2b, e, h. The shrunken patterns after each miniaturization step are shown in Fig. 2c, f, i. After the first miniaturization step, the pattern size was shrunken by 50%; the line width was reduced to 675 nm and the spacing to 320 nm. After the second step, the line width was reduced to 280 nm and the spacing to 190 nm, showing a 77% total reduction from the initial pattern. After the third miniaturization step, the line width was reduced to 130 nm, while the spacing was reduced to 100 nm, which resulted in an 89% total reduction compared to the initial pattern. The results of the multistep miniaturization approach show that from a single master pattern, three higher-resolution patterns were fabricated with high fidelity, achieving a feature size as small as 100 nm for the smallest pattern. It should be noted that the spacing between the lines was reduced more than the line width itself, probably because of the effect of the imprinting process. When the imprinted film is thermally heated, the compressive stresses embedded in the prestressed film are released, causing the polymer material to shrink to a smaller size. However, the imprinting process that creates the features also plastically deforms the material and introduces additional stresses at the surface in addition to the prestress in the bulk. During the imprint process, the material is moved from the spacing region into the line width region. This creates a large stress at the surface in the spacing compared with that in the line width region. Therefore, release of this stress by thermal shrinking can potentially shrink the spacing more than the line width. Since the stress-induced is dependent on the imprinting process and the amount of material displaced, one would expect that the asymmetry in the shrinkage will decrease as smaller and smaller patterns are imprinted. This is seen in the results obtained, where the asymmetry in the shrinkage is larger when the 1 µm patterns are imprinted than with smaller patterns. Here, the applied hot embossing conditions lead to a 50–55% shrinkage ratio instead of the 60% shrinkage ratio for typical shrinkable PS films^24–26^. Each miniaturization cycle takes ~3 h to complete. Therefore, a size reduction from 1 µm to ~100 nm can be accomplished over 3 cycles, which will take ~9 h. This compares favorably with direct write nanolithography methods such as e-beam, which takes ~24 hr for 1 cm^2^. It should be noted that the processing time for the multistep miniaturization process is not dependent on the pattern size or the complexity of the feature, unlike other direct write methods.

### Aspect ratio of the fabricated nanopillar array

**Fig. 3:**
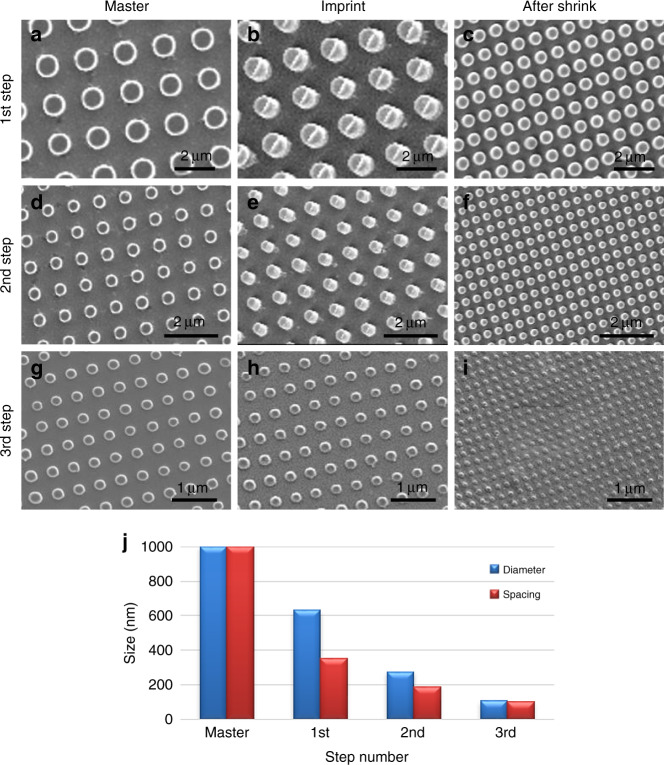
Multistep miniaturization of pillars array for three successive cycles. SEM images of **a**, **d**, **g** Si master, **b**, **e**, **h** imprinted prestressed film, and **c**, **f**, **i** shrunk pattern for each miniaturization step. **j** Graph of size reduction over miniaturization steps. The initial master pattern has pillar diameter *d* = 1 μm, spacing *s* = 1 μm. After shrinking of first step *d* = 630 nm, *s* = 350 nm, after second step *d* = 275 nm, *s* = 190 nm, after third step *d* = 105 nm, s = 100 nm

The multistep miniaturization process can be used for the nanofabrication of different feature shapes. In order to demonstrate the variety of features that can be miniaturized, a pillar array was fabricated. The initial master of the pillar array has circular pillars 1 µm in diameter with similar spacing between pillars (Fig. 3a). The initial pattern was imprinted and miniaturized for three sequential miniaturization cycles. The initial master and the following fabricated masters are shown in Fig. 3a, d, g. The imprinted patterns on PS films after hot embossing are shown in Fig. 3b, e, h. The miniaturized patterns after constrained shrinking are shown in Fig. 3c, f, i. After the first miniaturization step, the pillar diameter was reduced from 1 µm to 630 nm, while the spacing was reduced to 350 nm, showing an overall reduction of 51%. After the second step, the pillar diameter was reduced further to 275 nm and the spacing to 190 nm, achieving a 77% total reduction in size from the initial master. After the third miniaturization step, the pillar diameter was further reduced to 105 nm and the spacing to 100 nm, which resulted in a significant total reduction of 90% compared to the initial pattern. The proportional size reduction of the pillar array over three miniaturization steps is shown in Fig. 3j. Similar to the line-space pattern, the spacing between pillars shrank more than the pillar diameter due to the stress-induced during the imprinting process. However, the difference between pillar diameter and spacing decreased when the shrunken pattern was transferred to a Si substrate to fabricate a master for the next step. After patterning the UV-curable polymer mask on the Si substrate (Fig. 1f), the residual polymer layer was removed by a quick RIE using oxygen to expose the Si surface. During this RIE process, the polymer pattern features were also slightly etched in the lateral directions, which led to a reduction in the pillar diameter and an increase in the spacing between them. Then, the pattern was transferred to the Si substrate by selective RIE of Si. The results show that the pattern integrity was maintained and high-resolution patterns were fabricated with a 10× reduction in size from the original pattern.

**Fig. 4:**
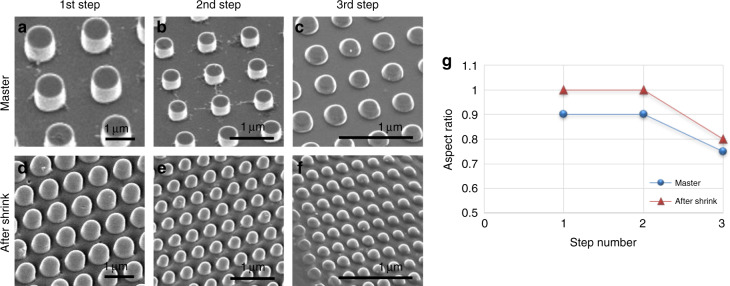
Aspect ratio of the fabricated patterns. SEM images of **a**–**c** Si master, and **d**–**f** shrunk patterns for three miniaturization steps. **g** Aspect ratio of the master and shrunk patterns for each miniaturization step

The multistep miniaturization approach is based on shrinking patterns on prestressed polymer films that were imprinted by hot embossing. However, direct shrinking of hot-embossed patterns dramatically decreases the height of the patterns, which consequently tend to disappear after shrinking (Supplementary Information, section 3). As a result, the aspect ratio is also dramatically reduced. In contrast, the developed constrained shrinking process allows a reduction in the pattern size without loss of topographical features. In particular, the height of the patterns is retained or slightly decreased when applying directional constraints during shrinking, while the in-plane feature size dramatically decreases. Thus, the aspect ratio is expected to increase, which is considered an advantage in nanofabrication. However, increasing the aspect ratio during successive steps can result in tall and weak structures that can prevent precise pattern imprinting. The intermediate step of transferring the patterns obtained from constrained shrinkage onto a Si master allows independent control of the height of the pattern and therefore the aspect ratio over several miniaturization cycles. Figure 4 shows the aspect ratio of the pillar array over three miniaturization cycles. For each cycle, SEM images were taken at an inclined view for the master patterns (Fig. 4a, b, c) and the shrunken patterns (Fig. 4d, e, f) in order to illustrate the height of the fabricated pillars. The aspect ratios of the master and shrunken patterns are shown in Fig. 4g. It can be seen that the aspect ratio of features on the shrunken films increased after constrained shrinking compared to the initial master for each miniaturization step. In addition, the aspect ratio was maintained for the first and second miniaturization cycles as the height of the patterns in the master was controlled by RIE of the Si intermediate master. However, the aspect ratio of the master and shrunken patterns of the third step was lower. This may be due to the unoptimized RIE process when fabricating the third step master at such small dimensions. The polymer mask was etched quickly, and hence deeper etching of the Si could not be achieved. However, in the future, a more resistant mask can be used, and the RIE process can be optimized to maintain the required aspect ratio^27–29^. Of note is that DRIE is a widely used process in integrated circuit nanofabrication, and high resolutions <50 nm are obtainable with good process control.

### Fabrication of complex patterns

**Fig. 5:**
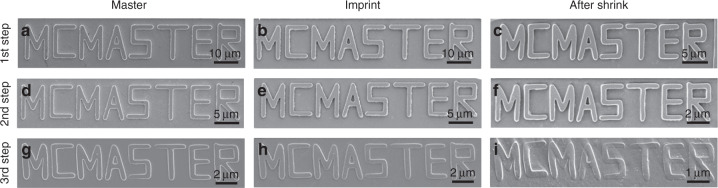
Multistep miniaturization of “MCMASTER” name for three successive cycles. SEM images of **a**, **d**, **g** Si master, **b**, **e**, **h** imprinted prestressed film, and **c**, **f**, **i** shrunk pattern for each miniaturization step

In order to demonstrate that the multistep miniaturization approach is versatile, a more complicated pattern of letters (MCMASTER) was also fabricated and miniaturized for three sequential miniaturization cycles (Fig. 5). The total reduction in size achieved after each miniaturization cycle was 51%, 78%, and 88%, respectively. The lines forming each letter of MCMASTER had an initial width of 2 µm. After each miniaturization step, the line width was reduced to 970, 450, and 240 nm in turn. It can be seen that all the letters were miniaturized in the same proportion, even the letters that include diagonal lines such as “A”. This indicates that the technique is versatile and can proportionally miniaturize not only line and dot patterns but any complex pattern of choice. It can be noted that the shrunken pattern after the third miniaturization step was slightly distorted (Fig. 5i). This may be due to misalignment in the constrained direction during the shrinking process. Such distortions can often occur when the constraints on either side are not aligned with each other. The use of a more optimized jig for the constraint would address this issue. Nevertheless, the results show that the multistep miniaturization approach can be used for almost any kind of patterns and for different feature shapes to significantly reduce the size of the original features from the micrometer scale to the nanoscale. This can overcome the challenges of directly fabricating nanopatterns using conventional photolithography methods.

The combination of constrained shrinking with multistep miniaturization allows patterns that are easily producible on a micrometer scale with optical lithography or other such methods to be reduced by at least an order of magnitude in dimension to the nanometer scale. The process is highly repeatable and consistent and is dependent only on the material property (amount of prestress embedded) of the film used. Furthermore, the process tools that are used, such as deep reactive ion-etching systems, are widely available and lower in cost than e-beam lithography systems. Due to its many advantages, this process has the potential to democratize the production of nanoscale patterns in facilities that may not have access to expensive tools such as e-beams or focused ion beam writers.

## Conclusion

In summary, we have demonstrated a multistep miniaturization approach using prestressed polymer films that can reduce the size of original patterns dramatically from the microscale to the nanoscale. Patterns imprinted on prestressed films by hot embossing were miniaturized using a constrained shrinking process that made it possible to reduce the size of the initial patterns without losing topographical features. The miniaturization process can be repeated several times for further size reduction by using the shrunken patterns to generate new masters for subsequent miniaturization steps. We have fabricated well-defined, high-resolution nanoscale patterns with sizes as small as 100 nm starting from a master with patterns 1 µm in size. From a single master, three smaller patterns were fabricated, achieving a 10× reduction in size over three miniaturization steps. This powerful approach can be used to fabricate masters with nanoscale features and overcome the challenges of nanofabrication using serial writing techniques. Moreover, we have shown that the aspect ratio of the miniaturized patterns can be maintained over several miniaturization cycles, which improves the pattern fidelity. The multistep miniaturization approach described here is a scalable and versatile nanofabrication process that can be used for a wide range of applications, including integrated circuit manufacturing by nanoimprint lithography. The ultimate achievable resolution is determined by the radius of gyration of the polymer and can be as small as 1–10 nm depending on the polymer chain length. Refinement of the processes, including the DRIE and the hot embossing, with tighter process control can allow this process to be extended to that limit and is important for its commercial implementation.

## Experimental

### Polymer working stamp fabrication

The working stamp was fabricated using a UV-curable polymer (MD700, Solvay) attached to a glass substrate. The glass substrate was first coated with an adhesion promoter (EVGprimK) to improve polymer adhesion to the glass surface. The working stamp was replicated from a Si master, which was fabricated by a direct laser writer (Heidelberg, µPG 101) to pattern a photoresist layer (S1808) coated on a Si substrate. Then, the Si was etched by an RIE, process and the remaining photoresist mask was removed. The Si master was coated with anti-stick layer before fabrication of the working stamp. The UV-curable polymer was mixed with photoinitiator (2% by weight) and placed in a vacuum chamber to outgas. Then, the polymer mixture was poured on the Si master, and the glass substrate was placed on top of them. The polymer was exposed to a UV light source for 5 min to cure. Finally, the working stamp was carefully separated from the Si master, and the excess polymer was rinsed with HFE solvent.

### Hot embossing of prestressed films

Polystyrene prestressed films (Graphix Shrink Film, Maple Heights, Ohio) were imprinted by hot embossing using (EVG520 HE) equipment. The fabricated polymer working stamp was used to imprint the prestressed films. The hot embossing process parameters were as follows: molding temperature 125 °C, molding force 4500 N, and molding time 5 min. After hot embossing, the imprinted film and polymer stamp were cooled while the molding force was still applied. Then, the imprinted film was carefully demolded from the stamp at a demolding temperature of ~60 °C.

### Constrained shrinking

After imprinting of the patterns onto the prestressed film, the film was mechanically constrained at two opposite ends along one direction using paper binder clips. The patterned area was placed in the middle between the two constrained ends. The assembly was placed in an oven and heated at 130 °C for 9–10 min. This resulted in shrinking the film in one direction only, orthogonal to the constrained direction. In order to obtain biaxial shrinkage, the film was then constrained in the orthogonal direction with the patterned area at the middle and heated again at the same temperature and time.

### Reactive ion-etching RIE

The shrunken patterns were transferred to a Si substrate by RIE to fabricate a master for the next miniaturization step. RIE equipment (Oxford PlasmaPro 100) was used. The mixed gases RIE process was used to etch Si patterns with the gases C4F8 and SF6 at flow rates of 100 sccm and 50 sccm, respectively. The RIE time was 1–2 min depending on the required etching depth. The SF6 flow rate was reduced to 40 sccm when etching the smaller patterns to improve the selectivity.

### SEM imaging

SEM images were taken using a JEOL JSM-7000F. The imprinted prestressed films were coated with a thin gold layer (8 nm) to prepare the samples for SEM imaging. The Si patterns were imaged without coating.

## Supplementary information

supplementary material

## References

[1] Sreenivasan S (2017). Nanoimprint lithography steppers for volume fabrication of leading-edge semiconductor integrated circuits. Microsyst. Nanoeng..

[2] Sebastian EM, Jain SK, Purohit R, Dhakad S, Rana R (2020). Nanolithography and its current advancements. Mater. Today Proc..

[3] van Assenbergh P, Meinders E, Geraedts J, Dodou D (2018). Nanostructure and microstructure fabrication: from desired properties to suitable processes. Small.

[4] Roeder M, Guenther T, Zimmermann A (2019). Review on fabrication technologies for optical mold inserts. Micromachines.

[5] Menon R, Patel A, Gil D, Smith HI (2005). Maskless lithography. Mater. Today.

[6] Chen J (2010). A versatile pattern inversion process based on thermal and soft UV nanoimprint lithography techniques. Microelectron. Eng..

[7] Lan H, Liu H (2013). UV-nanoimprint lithography: structure, materials and fabrication of flexible molds. J. Nanosci. Nanotechnol..

[8] Kapl SE (2011). Characterization of CMOS programmable multi-beam blanking arrays as used for programmable multi-beam projection lithography and resistless nanopatterning. J. Micromech. Microeng..

[9] Matsumoto, H. et al. Multi-beam mask writer MBM-1000 and its application field. In *Photomask Japan 2016: XXIII Symposium on Photomask and Next- Generation Lithography Mask Technology* 998405 (2016).

[10] Hu J, Zhu Y, Huang H, Lu J (2012). Recent advances in shape–memory polymers: Structure, mechanism, functionality, modeling and applications. Prog. Polym. Sci..

[11] Mather PT, Luo X, Rousseau IA (2009). Shape memory polymer research. Annu. Rev. Mater. Res..

[12] Lin S, Lee EK, Nguyen N, Khine M (2014). Thermally-induced miniaturization for micro-and nanofabrication: progress and updates. Lab a Chip.

[13] Nguyen D (2010). Better shrinkage than shrinky-dinks. Lab a Chip.

[14] Goodrich PJ, Sharifi F, Hashemi N (2015). Rapid prototyping of microchannels with surface patterns for fabrication of polymer fibers. RSC Adv..

[15] Grimes A (2008). Shrinky-Dink microfluidics: rapid generation of deep and rounded patterns. Lab a Chip.

[16] Zhao X-M, Xia Y, Schueller OJ, Qin D, Whitesides GM (1998). Fabrication of microstructures using shrinkable polystyrene films. Sens. Actuators A Phys..

[17] Sayed S, Selvaganapathy PR (2020). Multi-step proportional miniaturization to sub-micron dimensions using pre-stressed polymer films. Nanoscale Adv..

[18] Zhu X, Cui T (2013). Polymer shrinkage of hot embossed microstructures for higher aspect ratio and smaller size. Sens. Actuators A Phys..

[19] Sun J (2019). Development and application of hot embossing in polymer processing: a review. ES Mater. Manuf..

[20] Park S-M (2011). Sub-10 nm nanofabrication via nanoimprint directed self-assembly of block copolymers. ACS Nano.

[21] Yokoo, A., Wada, K. & Kimerling, L. C. Pattern size reduction of nanoprint-fabricated structures on heat-shrinkable film. *Japan. J. Appl. Phys.* **46**, 6395 (2007).

[22] Sayed S, Selvaganapathy PR (2021). Constrained shrinking of nanoimprinted pre-stressed polymer films to achieve programmable, high-resolution, miniaturized nanopatterns. Nanotechnology.

[23] Glinsner T (2010). Fully automated hot embossing processes utilizing high resolution working stamps. J. Vac. Sci. Technol. B Nanotechnol. Microelectron..

[24] Peng L, Deng Y, Yi P, Lai X (2013). Micro hot embossing of thermoplastic polymers: a review. J. Micromech. Microeng..

[25] Dyer D (2011). Sequential shrink photolithography for plastic microlens arrays. Appl. Phys. Lett..

[26] Gabardo CM, Zhu Y, Soleymani L, Moran‐Mirabal JM (2013). Bench‐top fabrication of hierarchically structured high‐ surface‐area electrodes. Adv. Funct. Mater..

[27] Baquedano E, Martinez RV, Llorens JM, Postigo PA (2017). Fabrication of silicon nanobelts and nanopillars by soft lithography for hydrophobic and hydrophilic photonic surfaces. Nanomaterials.

[28] Doll P (2019). Fabrication of silicon nanopillar arrays by electron beam lithography and reactive ion etching for advanced bacterial adhesion studies. Mater. Res. Express.

[29] Laermer, F., Franssila, S., Sainiemi, L. & Kolari, K. in *Handbook of Silicon Based MEMS Materials and Technologies* 417–446 (Elsevier, 2020).

