# Supplementary material for: High-resolution fabrication of nanopatterns by multistep iterative miniaturization of hot-embossed prestressed polymer films and constrained shrinking
Source: Microsyst Nanoeng. 2022 Feb 14;8:20. doi: 10.1038/s41378-021-00338-y (PMC8841498; doi:10.1038/s41378-021-00338-y)
Supplement: Supplementary file 1 — supplementary material [file 41378_2021_338_MOESM1_ESM.docx]

High resolution fabrication of nano patterns by multi-step iterative miniaturization of hot embossed pre-stressed polymer films and constrained shrinking

**Supplementary Information**

**1.Using polymer working stamp for hot embossing**


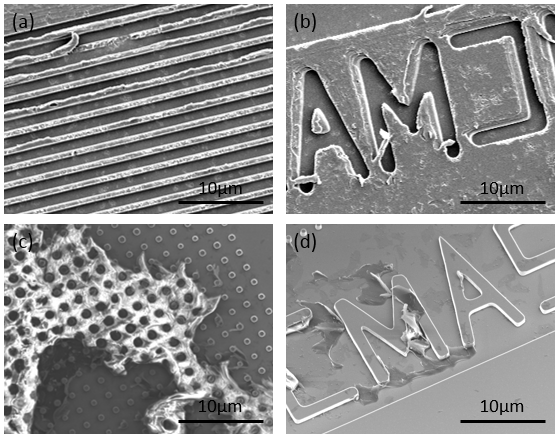
In the developed multi-step miniaturization approach a polymer working stamp which was replicated from the original Si master was used for hot embossing. However the Si master can be used directly for hot embossing, the polymer working stamp has several advantages over Si master. These advantages include: the polymer stamp is better in terms of demolding and repeatability, the imprinted pattern has same polarity as the original master, and increase lifetime of the master. The polymer working stamp was easier for demolding after hot embossing and did not have sticking issues as the Si master. Figure S1 illustrates demolding issues associated with using Si master directly for hot embossing. However an anti sticking layer was coated on the Si master before hot embossing, the imprinted patterns on the PS shrinkable film were damaged during demolding (figure S1a, b) due to sticking to the Si master. In addition, if the Si master is used to imprint another sample, it should be cleaned and coated again with anti sticking layer each time before hot embossing. When Si master was used for hot embossing twice without coating anti sticking layer before the second hot embossing, larger areas of the imprinted film stick to the Si master (figure S1c, d). However, the polymer stamp can be used for many times without further treatments.

Figure S1 Demolding issues of using Si master for hot embossing pre-stressed films. (a, b) Pre-stressed film after imprint using Si master, however Si master was coated with anti-stick layer. (c, d) Si master after hot embossing twice, large areas of polymer film stick to the Si master.

**2.Optimization of RIE**

The multi-step miniaturization approach allows using the shrunk pattern of a miniaturization cycle to fabricate a Si master that can be used for the next cycle. In order to transfer the pattern to fabricate a Si master, the Si substrate is etched through a RIE process. Different Si RIE recipes were used including Bosch process, mixed gases process, and a combination of both processes (the results are shown in figure S2). Bosch process which is a cyclic process of deposition/etch steps is used to obtain high aspect ratio structures. A protection layer which is generally a fluorocarbon-based film is deposited on the surface to protect sidewalls of the pattern. Then, the Si is etched at the bottom surface during the etch step while sidewalls are protected by the deposited layer. However, in our results, the deposited layer was insufficient to protect sidewalls which resulted in etching sidewalls completely (figure S2a). A combination of mixture gases etch followed by Bosch process was used (figure S2b, c, d). First, Si etch using gases mixture of C_4_F_8_/SF_6_ was performed which resulted in a good etch profile with smooth surface. Then, Bosch process was used to increase etch depth but it started to etch sidewalls as well (figure S2c). When Bosch process was performed for more cycles, the sidewalls were completely etched and the pattern was damaged (figure S2d). Thus, only mixture gases process was used to etch Si substrate for the desired depth with C_4_F_8_:SF_6_ gases ratio of 2:1 which resulted in good etch profile with smooth surface and nearly vertical sidewalls (figure S2e, f). This mixture gases RIE process was used for each miniaturization cycle in the multi-step miniaturization process.


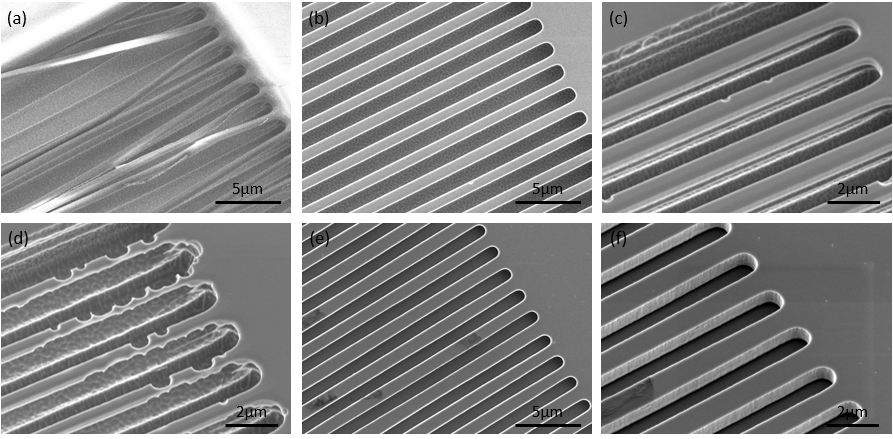


Figure S2 Si RIE using different processes. (a) Bosch process. (b, c) Mixed gases followed by Bosch process. (d) Mixed gases followed by Bosch process for longer time. (e, f) Mixed gases etch process.

**3.Direct shrinking of embossed pattern**


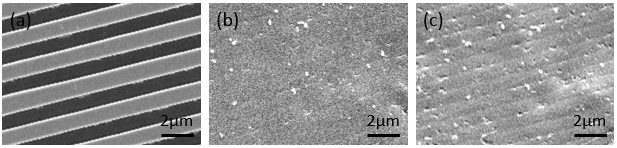
Direct shrinking of hot embossed pre-stressed films leads to loss of resolution as the height of the pattern dramatically decreased due to polymer reflow during shrinking (figure S3). The pre-stressed polymer film was imprinted by same hot embossing process, the imprinted pattern is shown in figure S3a. After direct shrinking, the pattern was almost disappeared (figure S3b). An inclined view for the shrunk pattern (figure S3c) shows that the pattern height is very small.

Figure S3 Direct shrinking of embossed pre-stressed film. SEM of (a) imprint, (b) shrunk pattern, (c) inclined view of the shrunk pattern.

**4.Fabrcating Si master instead of using shrunk PS film directly as a master for the next miniaturization step**


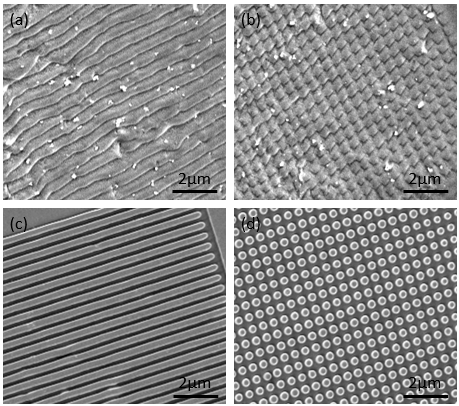
After thermal shrinking, the shrunk pattern was used to fabricate a new Si master which then was used as a master for the following miniaturization step. However the shrunk film could be used as a master directly, the results showed that the pattern had many limitations which prevent further successive miniaturization. After shrinking, the surface of the shrunk film was not completely flat compared to a Si substrate. Besides, the edges and sidewalls of the shrunk features become more curved due to softening of the polymer during heating. Thus, fabrication of an intermediate Si master allowed to generate precise patterns with vertical sidewalls which enabled continuing for further miniaturization steps. In order to demonstrate the limitations of using shrunk patterns directly for the next miniaturization cycle, the shrunk pattern of the first miniaturization cycle was used directly as a master for the second miniaturization cycle. The results of the second cycle after thermal shrinking are shown in figure S4 for both cases: using the shrunk film of the first step directly (figure S4a, b) and using the fabricated Si master (figure S4c, d). In the case that the shrunk film was used directly, the edges of the patterns became more curved and soft for both the line pattern (figure S4a) and pillars array (figure S4b) which resulted in losing the pattern fidelity. It can be seen also that the spacing between the features reduced significantly and the features were approximately in contact which resulted in closing the gaps between the features and merging them. In addition, the surface of the shrunk film was not completely flat which resulted in buckling and deformation of the patterns after shrinking particularly for the line pattern as shown in figure S4a. When an intermediate Si master was fabricated and used as a master for the second step, precise patterns were obtained after shrinking (figure S4c, d) similar to the shrunk patterns of the first step but at smaller dimensions. Thus, a Si master was fabricated and used as a master instead of the shrunk polymer film in the multi-step miniaturization process which allowed to overcome these limitations and improve the miniaturization process.

Figure S4 The results of second miniaturization cycle after shrinking (a, b) using the shrunk pattern of the first cycle as a master and (c, d) using the fabricated Si master.
